# Long‐term duration and safety of Radiesse (+) for the treatment of jawline

**DOI:** 10.1111/jocd.16436

**Published:** 2024-06-27

**Authors:** Jeremy B. Green, Roberta Del Campo, Alan J. Durkin, David K. Funt, Nicole Nasrallah, Keith Martinez, Amir Moradi

**Affiliations:** ^1^ Skin Research Institute and Skin Associates of South Florida Coral Gables Florida USA; ^2^ Del Campo Dermatology Miami Florida USA; ^3^ Ocean Drive Plastic Surgery and MedSpa Vero Beach Florida USA; ^4^ Florida State University College of Medicine Tallahassee Florida USA; ^5^ Icahn School of Medicine Mount Sinai Hospital New York New York USA; ^6^ Merz North America, Inc. Raleigh North Carolina USA; ^7^ Private Practice Vista California USA

**Keywords:** calcium hydroxylapatite, dermal filler, facial rejuvenation, jawline, volume restoration

## Abstract

**Background:**

Calcium hydroxyapatite (CaHA)‐carboxymethylcellulose (CMC)+ has unique properties that make it optimal for lifting, contouring, and defining the jawline. This long‐term follow‐up of a randomized, multicenter, rater‐blinded trial reports efficacy and safety of CaHA‐CMC(+) through 48 and up to 60 weeks post‐treatment.

**Methods:**

Eligible patients were randomized (2:1) to the treatment or the control/delayed treatment group to receive CaHA‐CMC(+) injections in both jawlines. While touch‐ups were permitted 4 weeks post‐treatment for both groups, only the treatment group was eligible for optional retreatment after 48 weeks. The primary outcome was ≥1‐point improvement on both jawlines on the Merz Jawline Assessment Scale (MJAS); secondary endpoints included the Subject Global Aesthetic Improvement Scale (SGAIS) among others. Post hoc analysis included pooling up to 48‐week data from the combined treatment and control/delayed groups and 60‐week data for the treatment group.

**Results:**

Overall, 175 received treatment. MJAS responder rates were 77.9%, 78.7%, and 62.9% at 12, 24, and 48 weeks post‐treatment, respectively. Responder rate on the MJAS at 60 weeks was 74.6% for those who received retreatment and 43.5% for those patients who received only the initial and touchup treatments. SGAIS scores demonstrated 93.4%, 85.6%, and 68.5% of patients rated themselves very much improved after 12, 24, and 48 weeks, respectively. Adverse events consisted of procedure or CaHA‐CMC(+)‐related events that were mostly resolved and overwhelmingly mild.

**Conclusions:**

CaHA‐CMC(+) produced clinically meaningful and long‐lasting improvements in jawline contour and was well tolerated in patients through 60 weeks.

**Trial Registration:**

ClinicalTrials.gov Identifier: NCT03583359.

## 1 INTRODUCTION

A contoured jawline contributes substantially to facial attractiveness. A taut or “snatched” jawline that clearly delineates the face from the neck is considered a powerful standard of beauty.^1^ The jawline, however, is particularly susceptible to bone resorption, jowl formation with scalloping of the jawline, soft tissue laxity (such as shortening of the mandibular length), and loss of soft tissue volume.^1^ These jawline changes can result in widening of the mandibular angle and loss of definition.^1^, ^2^ Thus, there is an increased interest in procedures and products that can improve jawline contour by sharpening the jawline and subsequent shadow on the neck, camouflaging the jowls, and creating volume thereby reducing laxity.

Minimally invasive modalities that improve facial contour and volume, such as dermal fillers, are among the more commonly used treatments to obtain aesthetic corrections.^3^ Calcium hydroxyapatite‐carboxymethylcellulose (CaHA‐CMC; Radiesse®; Merz North America, Inc., Raleigh, NC) is a fully biodegradable treatment that is dually‐endowed with direct filling and regenerative properties. CaHA‐CMC is composed of calcium hydroxylapatite microspheres suspended in a CMC‐based carrier gel. It is available in two formulations, CaHA‐CMC (Radiesse®) and CaHA‐CMC(+) (Radiesse® [+]; Merz North America, Inc., Raleigh, NC) which contains 0.3% lidocaine.^1^ CaHA‐CMC(+) is a high elastic and complex modulus product that is an effective nonsurgical option for redefining the mandibular angle and straightening the contour of the jaw.^4^ CaHA microspheres serve as scaffolds for dermal fibroblasts to stimulate the production of endogenous elastin, collagen types I and III, and increase proteoglycans and angiogenesis, which provides long‐term regeneration of multiple components of the extracellular matrix.^5^, ^6^, ^7^, ^8^, ^9^

In a prospective, multicenter, randomized, controlled, rater‐blinded pivotal trial (NCT03583359), the efficacy, and safety of CaHA‐CMC(+) was evaluated in adult patients with moderate or severe ratings on the Merz Jawline Assessment Scale (MJAS).^10^ The results of the primary analysis of this study performed at 12 weeks after treatment have been previously published.^10^ The objective of this analysis is to describe the long‐term results of this study through 48 and up to 60 weeks following initial treatment.

## 2 METHODS

### 2.1 Trial design

The methodology of this study, including key exclusion criteria, treatment techniques, and a description of outcome parameters, has been previously published.^10^ In brief, this was a randomized, prospective, multicenter (15 sites), rater‐blinded, and controlled pivotal trial. The study was approved by institutional review boards at all participating sites and was conducted in accordance with the standards for Good Clinical Practice and the ethical principles of the Declaration of Helsinki. Written informed consent was obtained from all patients prior to any study procedures.

### 2.2 Patients

Eligible patients included adult (22–65 years) males and females with symmetrical right and left jawline ratings of 2 or 3 (moderate or severe) on the MJAS. Key exclusion criteria included non–age‐related skin or fat atrophy in the midfacial and/or jawline regions or diagnosis of a connective tissue disorder; skin laxity and/or sun damage beyond what is typical of the patient's age; prior surgery on the jaw or in the jawline area, or a permanent implant or graft in the lower face and/or jawline area that could interfere with assessments of effectiveness; treatment with semipermanent dermal fillers in the lower face and/or jawline area in the past 5 years; receipt of porcine‐based collagen fillers or CaHA fillers in the lower face and/or jawline area within 24 months; and administration of hyaluronic acid products within the prior 12 months.

### 2.3 Treatments

Eligible patients were randomized (2:1) to treatment with CaHA‐CMC(+) or to control/delayed treatment. Patients were further randomized (1:1) to be treated with either needle or cannula. Patients randomized to treatment received an initial CaHA‐CMC(+) injection in both the right and left jawlines with reassessment at 4 weeks for the need of a touch‐up in one or both jawlines to achieve optimal correction. Patients in the treatment group were eligible to receive retreatment with CaHA‐CMC(+) at 48 weeks post initial treatment, prior to the end of study assessment at Week 60. Patients randomized to the control/delayed group remained untreated until the primary endpoint assessment at 12 weeks, at which time they were treated. These patients were then followed for 48 weeks through the end of the trial at Week 60. Presented here are the pooled long‐term results of all treated patients through 48 weeks post initial treatment and those in the treatment group for up to 60 weeks.

### 2.4 Assessments and endpoints

The primary outcome was based on the 5‐point MJAS scale that assesses the loss of volume and descent of the jowl and facial soft tissue with grades ranging from 0 (continuous jawline contour with no loss of volume) to 4 (extreme loss of jawline contour and loss of jawline volume). Clinical response was defined as ≥1‐point improvement on both jawlines compared to baseline according to the MJAS as assessed by a blinded trained evaluator at 12 weeks after initial injection. Other efficacy endpoints included were patient‐rated scores on the Subject rated Global Aesthetic Improvement Scale (SGAIS), and the FACE‐Q™ Satisfaction with Lower Face and Jawline Scale. Safety assessments included investigator‐ and patient‐reported adverse events over the course of the study.

### 2.5 Statistical methods

No power calculations were generated for these post hoc analyses. Statistical methodology for evaluating the primary endpoint (i.e., MJAS responder rates for treatment vs. control/delayed treatment 12 weeks after initial injection) is detailed in the primary efficacy manuscript.^10^ Results for MJAS response at later time points and for secondary efficacy parameters were descriptively summarized as the number and percent of patients who achieved the outcome. No imputation methods were used to fill in missing subject ratings or values, and only available ratings were used when calculating efficacy and satisfaction based on MJAS, SGAIS, and FACEQ. Fisher's Exact Test was used to compare MJAS and SGAIS ratings at each timepoint between moderate and severe subjects at baseline.

## 3 RESULTS

### 3.1 Patients

Of the 219 patients screened, 180 were enrolled and randomized (123 to treatment and 57 to control/delayed treatment), 175 received treatment and 139 completed the study. Of those who received treatment, a total of 122 patients were eligible for retreatment with 76 opting to receive retreatment at Week 48. Patients were primarily female (81.1%) and White (80.6%), with a mean (standard deviation) age of 55.3 (7.1) years (Table 1). In the treatment group, the Fitzpatrick skin type was I–III in 62.6% of patients and IV–VI in 37.4% of patients. The corresponding values in the control/delayed treatment group were 56.1% and 43.9%. Baseline MJAS scores in both the right and left jawlines were moderate in 52.8% of patients in the treatment group and 59.6% of those in the control/delayed treatment group, with the remaining patients having a severe MJAS score at baseline. Reasons for discontinuation were withdrawal by subject (*n* = 21), lost to follow‐up (*n* = 15), protocol violation (*n* = 1), and other (*n* = 4). Discontinuation rates were similar between the treatment group (23.6%) and control/delayed treatment group (21.1%).

**TABLE 1:** Demographics and baseline characteristics.

Characteristic	Treatment (*n* = 123)	Control/delayed treatment (*n* = 57)	Total (*N* = 180)
Female, *n* (%)	102 (82.9)	44 (77.2)	146 (81.1)
Mean age, years (SD)	55.5 (7.3)	55.0 (6.6)	55.3 (7.1)
Ethnicity, *n* (%)			
Hispanic/Latino	22 (17.9)	9 (15.8)	31 (17.2)
Not Hispanic/Latino	101 (82.1)	48 (84.2)	149 (82.8)
Race, *n* (%)			
White	103 (83.7)	42 (73.7)	145 (80.6)
Black/African American	14 (11.4)	10 (17.5)	24 (13.3)
Asian	5 (4.1)	5 (8.8)	10 (5.6)
American Indian/Alaska Native	1 (0.8)	0	1 (0.6)
Fitzpatrick skin type, *n* (%)			
I–III	77 (62.6)	32 (56.1)	109 (60.6)
IV–VI	46 (37.4)	25 (43.9)	71 (39.4)
MJAS score, left jawline^a^			
Moderate	65 (52.8)	34 (59.6)	99 (55.0)
Severe	58 (47.2)	23 (40.4)	81 (45.0)
MJAS score, right jawline^a^			
Moderate	65 (52.8)	34 (59.6)	99 (55.0)
Severe	58 (47.2)	23 (40.4)	81 (45.0)

Abbreviations: MJAS, Merz Jawline Assessment Scale; SD, standard deviation.

^a^ By blinded evaluator.

### 3.2 Treatment

The median volume of CaHA‐CMC(+) injected at baseline was 3.6 and 2.4 mL at touch‐up, which equates to fewer than four total syringes (1.5 cc per syringe). The median baseline volume injected among those with moderate MJAS score at baseline was 3.0 mL, with a median touch‐up dosage of 2.1 mL. For those with severe MJAS at baseline, the median volumes injected at baseline and touch‐up were 4.4 and 2.6 mL, respectively. The median volume injected among treatment group subjects receiving retreatment 48 weeks after initial injection was 2.8 mL (Figure 1).

**FIGURE 1:**
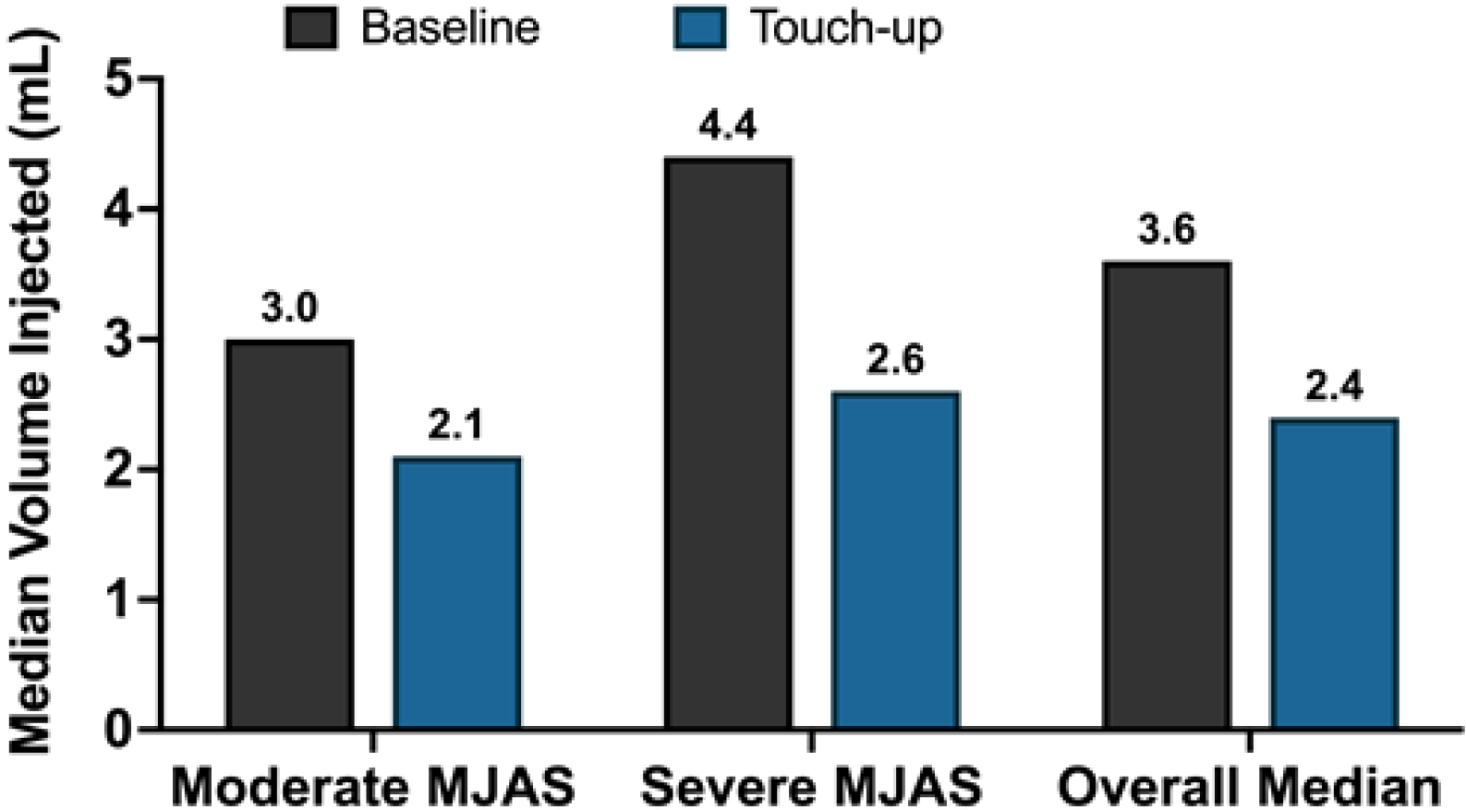
Median volume of CaHA‐CMC(+) injected at baseline and at touch‐up for moderate MJAS patients, severe MJAS patients, and all patients.

The most common injection techniques were serial puncture when injecting with a needle and linear‐threading/tunneling when injecting with a cannula. Injections using a needle were generally both supraperiosteal and subdermal, and cannula injections were most frequently subdermal.

### 3.3 Efficacy

Persistent improvement on the MJAS was evident for the duration of the follow‐up period with a responder rate of 77.9% (*n* = 134/172) at 12 weeks, 78.7% (*n* = 118/150) at 24 weeks, and 62.9% (90/143) at 48 weeks after initial injection. Of patients in the treatment group (who were eligible for retreatment), 74.6% (*n* = 53/71) of those retreated retained MJAS response at 60 weeks post initial injection and 43.5% (*n* = 10/23) of those not retreated retained response (Figure 2A). Response was achieved regardless of MJAS severity at baseline. Representative patient photographs before and after treatment are shown in Figure 3.

**FIGURE 2:**
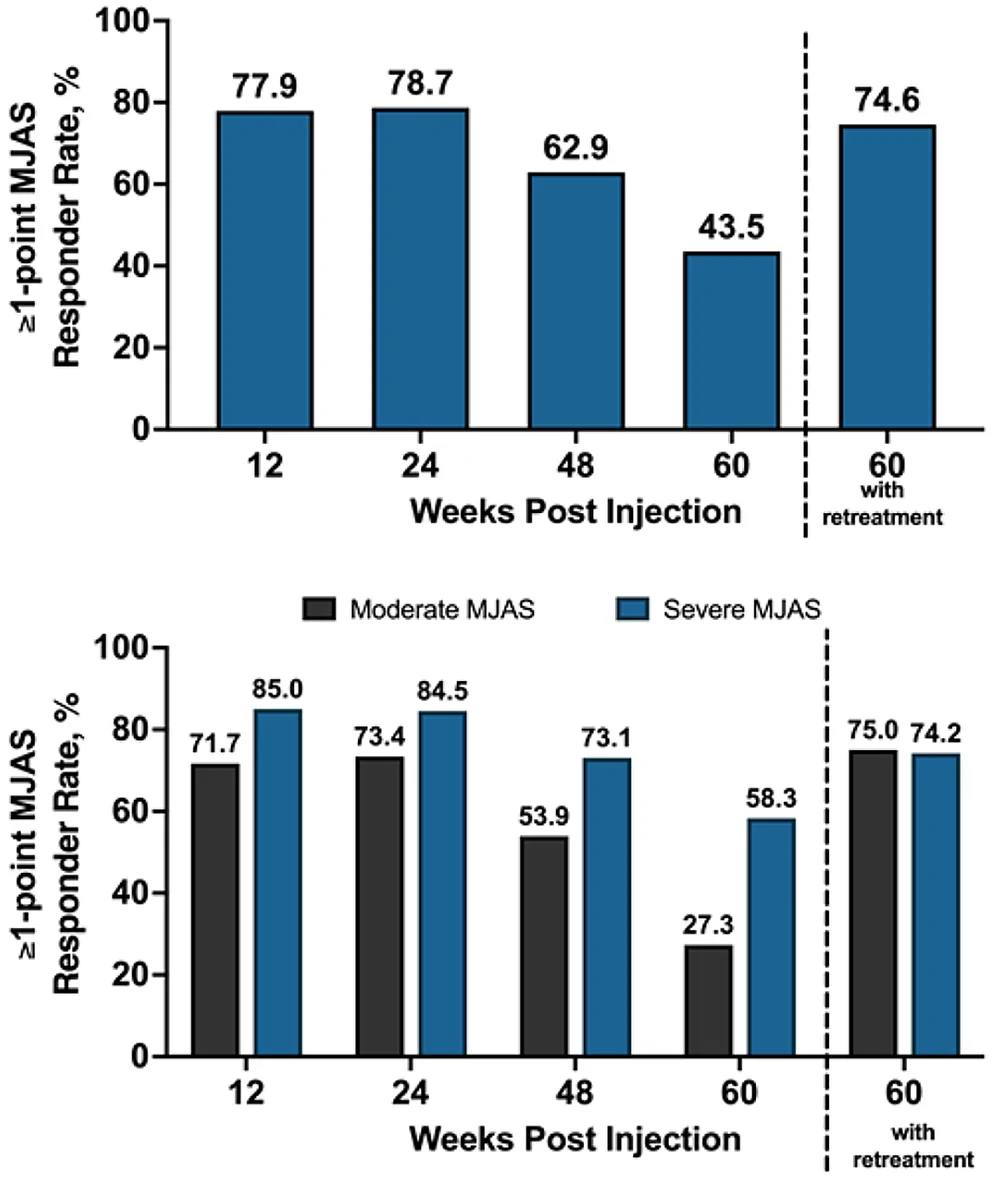
Response rates on the MJAS scale. (A) MJAS responder rates and (B) MJAS responder rates by MJAS severity. 60‐week results are from the treatment group only unless specified “with retreatment”. Responder = ≥1‐point improvement on both jawlines compared to baseline according to the MJAS. MJAS, Merz Jawline Assessment Scale.

**FIGURE 3:**
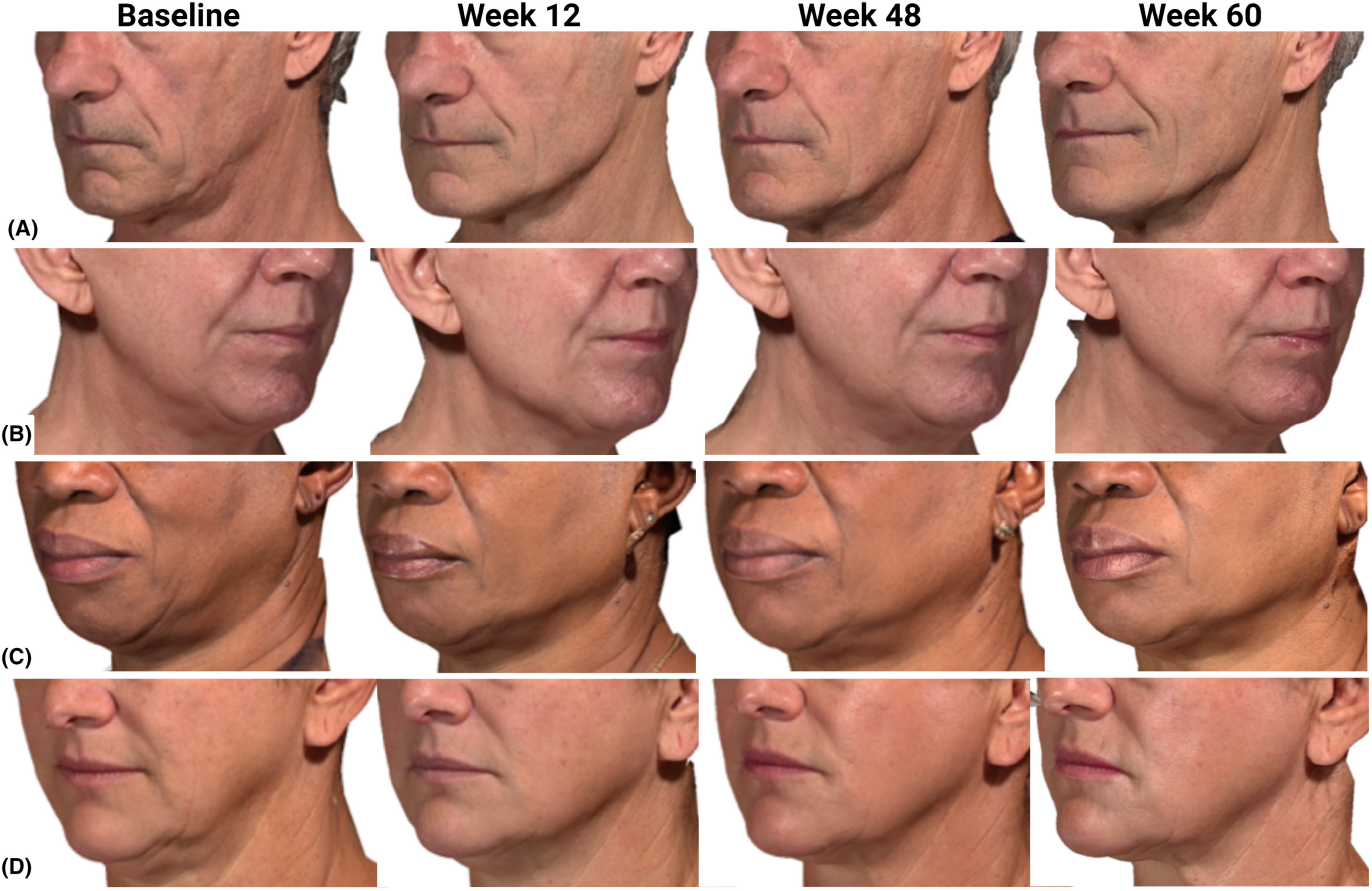
Representative photographs of the jawline at baseline, 12, 48, and 60 weeks after treatment with CaHA‐CMC(+). (A) Male, Fitzpatrick III, MJAS severe at baseline, total initial, and touch‐up volumes: 5.2 mL; (B) female, Fitzpatrick III, MJAS severe at baseline, total initial, and touch‐up volumes: 5.8 mL; (C) female, Fitzpatrick VI, MJAS severe at baseline, total initial, touch‐up, and retreatment volumes: 8.3 mL; (D) female, Fitzpatrick IV, MJAS moderate at baseline, total initial, touch‐up, and retreatment volumes: 4.9 mL.

Among patients with moderate MJAS at baseline, 71.7% (*n* = 66/92), 73.4% (*n* = 58/79), and 53.9% (*n* = 41/76) of patients retained treatment response at 12, 24, and 48 weeks after initial injection. Of those eligible for retreatment (treatment group) 75.0% (*n* = 30/40) retained treatment response among those retreated and 27.3% (*n* = 3/11) retained response among those not retreated at 60 weeks post initial injection (Figure 2B). For those with severe MJAS at baseline, treatment response was retained in 85.0% (*n* = 68/80), 84.5% (*n* = 60/71), and 73.1% (*n* = 49/67) at 12, 24, and 48 weeks, respectively (Figure 2B). At 60 weeks, treatment response was retained in 74.2% (*n* = 23/31) among those retreated and in 58.3% (*n* = 7/12) among those not retreated. No significance was found between moderate and severe MJAS response rates at each timepoint.

Treatment resulted in high satisfaction scores as evident by the change in the SGAIS from baseline, 93.4% (*n* = 156/167) of patients rated themselves as improved to very much improved 12 weeks after initial treatment. At 24 and 48 weeks after initial injection, 85.6% (*n* = 131/153) and 68.5% (*n* = 98/143) of patients felt improved to very much improved, respectively (Figure 4A). Among those eligible for retreatment, 82.2% (*n* = 60/73) of those retreated and 56.5% (*n* = 13/23) of those not retreated had improvement 60 weeks post initial injection.

**FIGURE 4:**
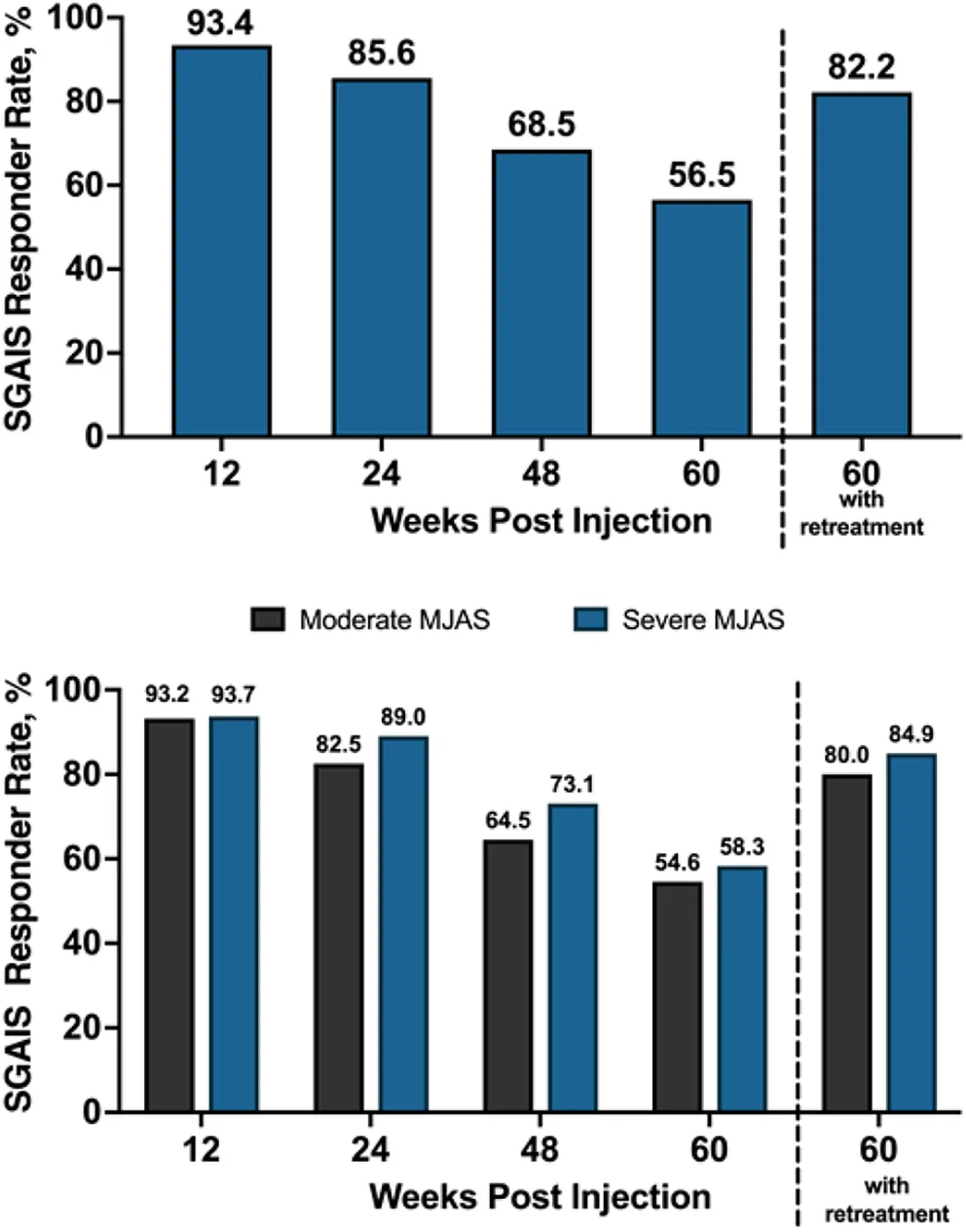
Responder rates on the SGAIS scale. (A) SGAIS responder rates and (B) SGAIS responder rates by MJAS severity. 60‐week results are from the treatment group only unless specified “with retreatment”. Responder = Improved, much improved, or very much improved from baseline. SGAIS, Subject rated Global Aesthetic Improvement Scale.

When stratified by MJAS severity at baseline, 93.2% (*n* = 82/88), 82.5% (*n* = 66/80), and 64.5% (*n* = 49/76) of patients with moderate MJAS had improved to very much improved SGAIS from baseline at 12, 24, and 48 weeks, respectively. Among those eligible for retreatment, 80.0% (*n* = 32/40) of those retreated and 54.5% (*n* = 6/11) of those not retreated had improvement 60 weeks post initial injection (Figure 4B). More patients with severe MJAS at baseline felt they had improved to very much improved, with 93.7% (*n* = 74/79) at 12 weeks, 89.0% (*n* = 65/73) at 24 weeks, and 73.1% (*n* = 49/67) at 48 weeks. Among severe patients eligible for retreatment, 84.9% (*n* = 28/33) of those that were retreated and 58.3% (*n* = 7/12) of those who were not retreated had improvement 60 weeks. No significance was found between moderate and severe SGAIS improvement rates at each timepoint.

For the FACE‐Q Satisfaction with Lower Face and Jawline assessment, the mean (standard deviation) Rasch‐transformed scores in patients increased from 21.7 (18.9) at baseline to 73.3 (22.4) at 12 weeks, 66.4 (25.9) at 24 weeks and 57.7 (28.0) at 48 weeks (Figure 5A). In the treatment group, the mean Rasch‐transformed score increased to 68.5 (24.5) among those retreated and 53.5 (32.1) among those not retreated 60 weeks post injection. For those with moderate MJAS at baseline, mean Rasch‐transformed scores increased from 23.3 (20.1) at baseline to 71.6 (20.3) at 12 weeks, 66.3 (25.6) at 24 weeks, and 56.6 (29.1) at 48 weeks (Figure 5B). Sixty weeks after initial injection, mean Rasch‐transformed scores were 66.7 (24.7) and 50.3 (34.2) for those retreated and not retreated respectively. For patients with severe MJAS at baseline these values increased from 19.9 (17.5) at baseline to 75.2 (24.6) at 12 weeks, 66.6 (26.4) at 24 weeks, and 58.9 (26.3) at 48 weeks. In the treatment group, patients retreated and not retreated had scores of 70.6 (24.4) and 56.8 (31.0), respectively. Overall, at 12, 24, and 48 weeks post injection, 96%, 91%, and 84% of all treated patients had improvement on the FACE‐Q from baseline (Figure 6A). In the treatment group, patients retreated and not retreated had 95% and 75% improvement from baseline, respectively. When stratifying improvement by MJAS severity, no significance in improvement was found at each timepoint (Figure 6B).

**FIGURE 5:**
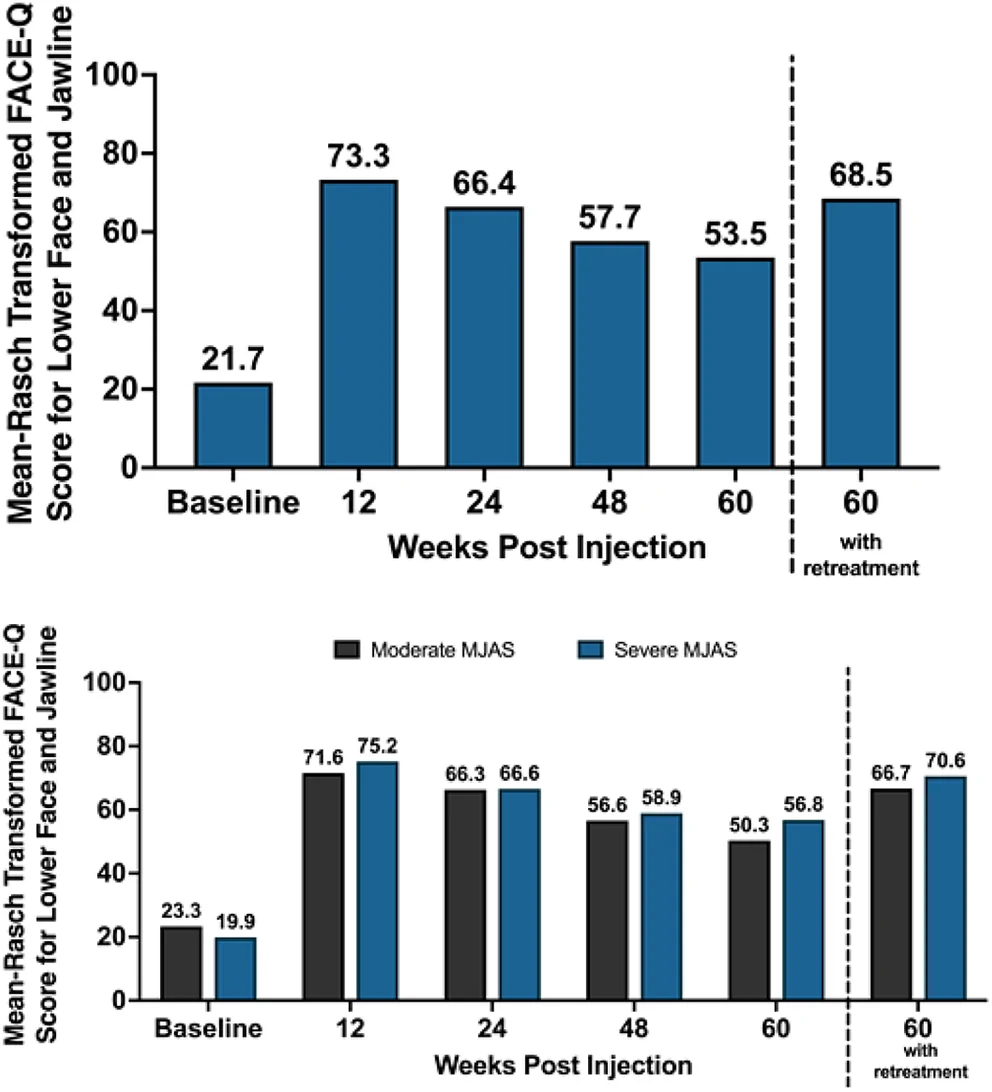
Mean Rasch‐transformed FACE‐Q satisfaction with lower face and jawline scores. (A) Mean Rasch‐transformed FACE‐Q Scores. (B) Mean Rasch‐transformed FACE‐Q Scores by MJAS severity. 60‐week results are from the treatment group only unless specified “with retreatment”.

**FIGURE 6:**
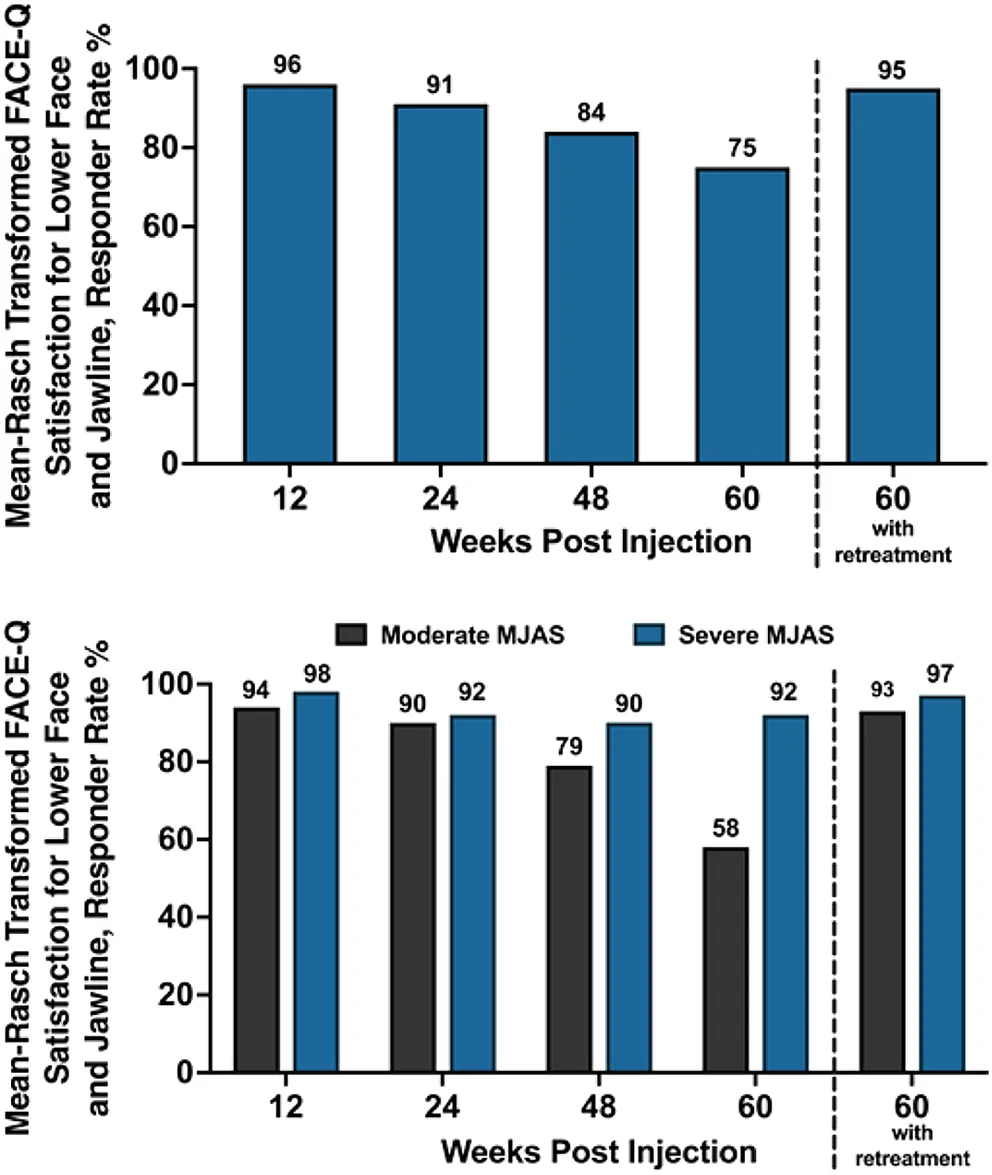
FACE‐Q proportion of subjects with improvement from baseline. (A) Proportion of subjects with improvement from baseline. (B) Proportion of subjects with improvement from baseline by MJAS severity. 60‐week results are from the treatment group only unless specified “with retreatment”.

At the end of the study (or at the early discontinuation visit), 77.2% (*n* = 112/145) of patients reported being extremely likely, likely, or somewhat likely to undergo re‐treatment.

### 3.4 Safety

CaHA‐CMC(+) was very well tolerated during the study and long‐term follow‐up period. Among all patients exposed to treatment (*N* = 175), 26.3% of patients had ≥1 treatment‐emergent adverse event (TEAE) that was considered related to either the injection procedure or to CaHA‐CMC(+) by the investigator. TEAE such as injection site bruising, erythema, edema, hemorrhage, mass, pain, rash, and swelling that were deemed only related to procedure occurred in a total of 21 patients. All TEAE related to only procedure were mild in nature with 64.3% resolved within ≤7 days and 92.9% resolved within ≤15 days without sequelae. TEAE related to only CaHA‐CMC(+) occurred in 13 patients and included injection site induration, inflammation, mass, nodule, and product distribution issue. All but one TEAE related to only CaHA‐CMC(+) were mild in nature with 40.0% resolved within ≤15 days and 60.0% resolved within ≤30 days. Overall, 28 patients had ≥1 TEAE believed to be related to both CaHA‐CMC(+) and the injection procedure. TEAE included injection site nodule, mass, redness, swelling, bruising, hemorrhage, and edema. All but two were mild in nature with 50.0% resolved within ≤7 days and 64.6% resolved within ≤15 days.

There were three serious TEAEs (1 CaHA‐CMC(+); two control/delayed treatment) consisting of cholecystitis, erysipelas, and hypertension, none were considered related to treatment or injection procedure. There were no TEAE that led to discontinuation from the study.

## 4 DISCUSSION

The long‐term follow‐up of this study demonstrate that CaHA‐CMC(+) provides a long duration of effect providing contour and volume to the jawline in patients with moderate to severe ratings on the MJAS at baseline. At 48 weeks post initial injection, approximately two thirds of patients retained MJAS treatment response and nearly three‐fourths were improved to very much improved on the SGAIS patient satisfaction scale. Notably, nearly half of the treatment group patients who received only the initial and touchup treatments maintained a clinically meaningful response out to 60 weeks and over half reported improvement on the SGAIS. SGAIS and patient satisfaction scores were generally higher in patients with severe MJAS at baseline compared to those with moderate MJAS at baseline. The results extend the initial 12‐week results, which found three‐fourths of patients achieving MJAS response.^10^ These long‐term results are in line with those of Bass et al.,^5^ who reported that the use of CaHA‐CMC was associated with prolonged efficacy for the treatment of nasolabial folds with some degree of improvement was seen up to 39 months post‐treatment.

CaHA‐CMC(+) has a variety of properties that contribute to sustained efficacy. CaHA‐CMC(+) has a sufficiently high complex moduli to directly fill and support surrounding soft tissue and prevent deformation under applied pressure.^11^ CaHA‐CMC and CaHA‐CMC(+) are unique in that they provide a dual mechanism of action. CaHA microspheres and the CMC gel provide immediate volume, while the interaction of the CaHA microspheres with fibroblasts produce an ongoing regenerative process.^9^, ^10^ Such interaction of CaHA microspheres with fibroblasts not only stimulate them to produce endogenous elastin and collagen types I and III,^6^, ^7^, ^12^, ^13^, ^14^ but also increase proteoglycans, angiogenesis, and neovascularization.^15^, ^16^, ^17^ Preclinical data suggest that CaHA‐CMC microspheres can reorganize collagen I fibers into a more highly organized and firm network similar to that of younger skin^13^ and that increased collagen can be observed for up to 78 weeks after injection.^12^ Neocollagenesis and neovascularization are thought to be important mechanisms associated with improvements in skin quality, roughness, and pigmentation.^9^, ^15^ Proteoglycans support the hydration of the skin and provide increased structural support in conjunction with collagen.^9^, ^16^ These histologic changes are accompanied by improvements in morphologic and mechanical properties of skin treated with CaHA.^9^ This includes a firmer and denser collagen network,^13^ increased dermal thickness, decreased wrinkle height and skin roughness, restoration of the functional properties of damaged fibroblasts, increased skin elasticity and plasticity, and overall improved appearance.^8^, ^15^, ^18^, ^19^ Thus, CaHA‐CMC(+) can potentially provide what other fillers cannot–the properties of an ideal facial filler^9^, ^20^ and a regenerative aesthetic treatment that can also clinically improve aging facial skin by restoring the native structure and overall mechanical properties of healthy skin.

In this study, CaHA‐CMC(+) was very well tolerated, with 20% patients experiencing TEAE that were considered related to the product. Injection site reactions were the most common TEAE experienced by just over 25%. All but three TEAEs were considered mild. There were no serious TEAEs deemed related to treatment and there were no treatment‐related discontinuations.

## 5 CONCLUSION

Overall, these results demonstrate that CaHA‐CMC(+) is a treatment that safely and effectively produces lift through 48 weeks post injection. Furthermore, CaHA‐CMC(+) may provide long‐term regeneration of the jawline, as seen in over 65% of patients with retained jawline improvement for over a year. Treatment with CaHA‐CMC(+) resulted in long‐term, clinically‐meaningful outcomes and a high level of patient satisfaction in patients with moderate to severe loss of jawline contour.

## AUTHOR CONTRIBUTIONS

J.B.G., R.D.C., A.J.D., D.K.F., N.N., K.M., and A.M. have read and approved the final manuscript. J.B.G., R.D.C., A.J.D., D.K.F., K.M., and A.M. conceived of the study design. J.B.G. and A.M. performed the clinical research. R.D.C., A.J.D., D.K.F., and K.M. interpreted the data and edited the manuscript. N.N. analyzed the data and edited the manuscript.

## Data Availability

Data available on request from the authors.
